# Subclinical atherosclerosis burden in carotid and femoral territories in HIV subjects: relationships with HIV and non-HIV related factors

**DOI:** 10.1186/s12879-024-09850-8

**Published:** 2024-09-09

**Authors:** Pedro Ferrer, Laura López, Juncal Pérez, Noemi Cabello, María José Núñez, Iñigo Sagastagoitia, Manuel Cotarelo, Leopoldo Pérez de Isla, Vicente Estrada

**Affiliations:** 1 Medical Affairs Department, MSD, Madrid, Spain; 2 Internal Medicine Unit, Hospital Clínico San Carlos, Madrid, Spain; 3 Cardiac Imaging Unit, Hospital Clínico San Carlos, Madrid, Spain

**Keywords:** HIV infection, Subclinical atherosclerosis, Vacular Elastography

## Abstract

**Background:**

Cardiovascular disease is a major cause of morbidity in an aging HIV population. However, risk estimation with the most frequent equations usually classifies HIV patients as having a low or moderate risk. Several studies have described a very high prevalence of subclinical atherosclerosis in a middle-aged, non-HIV population. There is insufficient body of knowledge to understand if this is the case in people living with HIV (PLWH). We aim to calculate the proportion of patients with subclinical atherosclerosis in a single site cohort of HIV-infected subjects.

**Methods:**

We have analyzed chronically HIV infected adults (≥ 18 years) who were on active follow-up in an HIV unit specialized in the care of cardiovascular health. The most recent clinical visit and vascular ultrasonography were used to assess the objectives of our research. Our primary objective was to describe the proportion of participants with subclinical atherosclerosis (focal protrusion into the lumen > 0.5 mm or > 50% of the surrounding IMT or a diffuse thickness > 1.5 mm) in a single site cohort of PLWH. Carotid and iliofemoral territories were evaluated. As a secondary objective we have run a multivariate analysis to determine which HIV and non-HIV factors might be related with the presence of atherosclerotic plaques.

**Findings**

We included a total of 463 participants between November 2017 to October 2019. Subjects were predominantly male (84.2%) with a mean age of 48.8 years (SD 10.7). Hypercholesterolemia (36%) was the most prevalent comorbidity followed by Hypertension (18%) and Hypertriglyceridemia (16%). Mean duration of HIV infection is 12.3 years. Overall, participants had been receiving cART for a median of 9.5 years. Subclinical atherosclerosis was found in 197 subjects (42.5%; CI 95% [38.0–47.2]). The disease was found more frequently in the femoral arteries (37.8%) than in the carotid vascular bed (18.6%). Despite some HIV factors correlated with the presence of plaques in a univariate analysis (e.g., time with HIV-1 RNA > 50 copies/mL or time from HIV diagnosis), the only two explanatory factors that remained associated with the presence of atherosclerotic plaques in the multivariate analysis were smoking (OR 5.47, 95% CI 3.36 – 8.90) and age (OR 1.13, 95%CI 1.10 – 1.16).

**Interpretation**

We have found a very high prevalence of subclinical atherosclerosis among our cohort of PLWH. Despite having analyzed several HIV factors, age and smoking have been found to be the only factors associated with the development of atherosclerotic plaques.

## Introduction

The advent of combined antiretroviral therapy (cART) has significantly changed the prognosis of people with HIV infection [1]. Besides reducing the number of AIDS related complications, such as opportunistic infections or HIV-associated tumors, cART has narrowed the gap of overall survival between HIV-infected people and the uninfected general population [2, 3]. However, there are still differences in the life expectancy of people living with HIV (PLWH) when compared with the uninfected population of similar characteristics [4]. This difference is mainly due to a higher prevalence of comorbidities in PLWH. HIV subjects do suffer more frequently ageing associated diseases such as atherosclerosis, cognitive impairment, osteoporosis, or chronic kidney disease among others [5–9].

Putting the focus in cardiovascular disease (CVD), PLWH do present it more often than does the general population with the same characteristics [10, 11]. A higher prevalence of traditional cardiovascular risk factors (CVRF) in this population may partially explain this finding [12–14]. However, even when traditional CVRF are similar, there is still an increased risk in PLWH. It is possible that chronic inflammation and immune activation in these persons could justify the difference [15].

The current body of knowledge suggests that the chronic inflammation and immune activation associated to HIV disease, may promote the mechanisms that lead to atherosclerotic plaque formation, thus increasing the risk of CVD in these individuals [16, 17]. However, an investigation carried out in a group of subjects resembling the general population, a very high prevalence (71% in men and 48% in women) of subclinical atherosclerosis has also been described in a non-HIV setting [18]. Other studies have shown similar results (AWHS) [19]. Whether the prevalence of subclinical atherosclerosis is even higher in PLWH is unclear. Based on the experience of these studies, vascular ultrasound techniques provide us with a non-invasive method to detect this subclinical disease in our population of PLWH. In addition, the guidelines from the European Society of Cardiology recommend using vascular ultrasonography to inform treatment choices in subjects that otherwise might have a low to moderate estimated risk [20]. Our initial hypothesis is that the prevalence of subclinical atherosclerosis would be higher than it was in the general population. If this is not true, we will need to explore alternative explanations for a higher prevalence of cardiovascular disease among the HIV population. These explanations could be more oriented to an impaired biology of plaque formation and stabilization leading to plaques more prone to rupture.

We, therefore, are aiming to estimate the proportion of participants with subclinical atherosclerosis in our cohort of PLWH. A multivariate analysis will be set up to identify HIV and non-HIV related factors that may be implicated in the generation of atherosclerotic plaques thus, giving clues for future interventions.

## Methods

### Study population and design

The *Vascular Assessment by Ultrasound in HIV Persons* (VASI study) is a single site, observational, cross-sectional research with partial retrospective data collection. Observation period was equal or before the date of the participant inclusion in the study. All participants were recruited in the HIV unit of Hospital San Carlos in Madrid where a total of 2,500 persons are followed. As part of their routine care, we evaluate the cardiovascular health of these persons every six months as part of a monographic visit in the unit. We aimed to recruit 577 consecutive persons what will provide a statistical precision < 4% in the estimation of the population proportion with asymptotic normal 95% confidence interval and assuming a prevalence of subclinical atherosclerosis > 60%.

We recruited chronically infected HIV-1 persons 18 years old or older. Participants should have had their last cardiovascular assessment performed within the last 8 weeks and the last vascular ultrasonography performed within the last 24 months. We excluded people with acute HIV-1 infection or with established cardiovascular disease at the time of HIV-1 diagnosis.

### Study variables and outcomes

After completion of a regular visit in our HIV unit study participants were invited to participate in the VASI study. Individual clinical records were used as the source for both non-HIV and HIV characteristics that should have been collected either the same day of consent or within the last 8 weeks. Non-HIV key characteristics collected for the study purposes included demographic characteristics (age, gender, race, BMI and blood pressure), traditional cardiovascular risk factors (arterial hypertension, smoking habit, exercise habit, total cholesterol and its fractions and diabetes diagnosis) and history of comorbidities (e.g., chronic kidney disease, metabolic complications, bone disease, liver disease, psychiatric conditions etc.). An indicator of HIV disease severity and risk of all-cause mortality after one year of cART was also calculated (The Veteran Aging Cohort Study -VACS- Index) [21]. Cardiovascular risk was calculated with four different equations (Score, D:A:D risk equation, Regicor [22] & Framingham). Participants were classified as active smokers if they smoke a minimum of seven cigarettes weekly or if they quit smoking less than one year ago. HIV characteristics collected for the study purposes included date of HIV diagnosis, HIV risk category, cumulative time with HIV-1 RNA above 50 copies per mL, current levels (and percentage) of CD4 and CD8 cells, current CD4/CD8 ratio, CD4 nadir, ART history for the last 10 years and CDC HIV stage.

If available from the same clinical visit than the vascular study, the following inflammation and coagulation markers were analyzed to describe their relationship with the presence and extension of subclinical atherosclerosis: high-sensitivity C reactive protein (hsCRP), interleukin 6 (IL-6), D-dimer, soluble cluster of differentiation 14 (CD14) and soluble cluster of differentiation 163 (CD163).

The last vascular ultrasound available from each person was used to determine the presence of atherosclerotic plaques in four vascular beds (right and left carotid and ileo-femoral arteries).

Vascular 2-dimensional echography were performed in the different vascular territories. A conventional ultrasound device equipped with a linear probe specifically dedicated to vascular studies were used. The same device and the same probe were used in every participant. Images were stored in DICOM format and they were analyzed off-line by the same expert reader.

Presence of atherosclerotic plaque was defined as a focal protrusion into the lumen > 0.5 mm or > 50% of the surrounding IMT or a diffuse thickness > 1.5 mm. Additionally atherosclerotic plaques were characterized by its echogenicity, texture and calcification.

Echogenicity of the plaques were classified as low (high level of lipid content and inflammatory activity), moderate (intermediate situation) or high (calcification and fibrosis). Texture of the plaques were classified as homogeneous (only one ultrasonographic density in the plaque) or heterogeneous (two or more ultrasonographic densities in the plaque). The mechanism of plaque calcification was considered as present if calcium was detected in at least one plaque in each patient.

Extension of the disease was also analyzed and graded from 0 (no disease present) to 4 (all vascular territories affected).

### Statistical analysis

We used descriptive statistics to show the characteristics of the population under study. Prevalence of subclinical atherosclerosis has been calculated as the proportion of participants with at least one vascular bed affected divided by the total number of participants analyzed. Point estimates and 95% CI has been used in the estimation of the main parameter.

A single multivariate binary logistic regression model has been used to identify the factors (HIV and non-HIV related) associated with the presence of atherosclerotic plaques.

The presence or absence of atherosclerotic plaques were used as a binary response variable.

Factors that were significant in bivariate analysis versus the response variable has been considered in a priori selection of potentially explanatory factors to be initially included in the model. In the former analyses, a level of significance < 0.10 were considered.

Explanatory variables highly correlated with each other were not included in the model. In the presence of two or more explanatory variables highly correlated, only one variable was selected to enter in the model to avoid issues related to collinearity.

## Results

### Characteristics of study participants

At selection, 529 persons consented to participate. Sixty-six of these persons were excluded of the final analysis because some of the selection criteria were not met, resulting in a total of 463 evaluable participants. A summary of the demographic characteristics as well as the comorbidities present in this cohort are listed in Table 1. Participants were predominantly male (84.2%) with a mean age of 48.8 years (SD 10.7). Hypercholesterolemia (36%) was the most prevalent comorbidity followed by hypertension (18%) and hypertriglyceridemia (16%).

**Table 1:** Demographics, comorbidities and HIV Characteristics

*Demographics*	TOTAL (*N* = 463)
Male gender, *n* (%)	390 (84.2)
Age (years), mean ± SD	48.8 ± 10.7
Caucasian ethnicity, *n* (%)	405 (87.5)
BMI, mean ± SD (Kg/m^2^)	24.7 ± 3.6
Systolic blood pressure, mean ± SD (mmHg)	122.9 ± 13.2
Diastolic blood pressure, mean ± SD (mmHg)	75.7 ± 8.7
***Comorbidities; n (%)***	
Hypertension	83 (18)
Diabetes Mellitus	27 (6)
Hypercholesterolemia^a^	166 (36)
Hypertrigliceridemia	75 (16)
Chronic Kidney Disease	16 (3.5)
***HIV Characteristics***	
HIV disease status, *n* (%)	
Stage 1	126 (27)
Stage 2	206 (44.5)
Stage 3	115 (25)
Unknown	16 (3.5)
HIV transmission risk factor, *n* (%)	
- Heterosexual sex	57 (12.3)
- Male who have sex with men	230 (49.7)
- Intravenous drug users	90 (19.4)
- Unknown	86 (18.6)
HIV-1 RNA concentration below limit of detection, *n* (%)	457 (99)
CD4 cell count (cells /µL), median (Q1 – Q3)	
Current	701 (522 – 941)
Nadir	289 (149–449)
Current CD8 cell count (cells /µL), median (Q1 – Q3)	865 (652–1,149)
Current CD4/CD8 ratio, median (Q1-Q3)	0.8 (0.6 – 1.1)
Antiretroviral history	
Time on cART, median (Q1-Q3); years	9.5 (4.9–11.8)
Time on NRTI, median (Q1-Q3); years	7.2 (4.2–11.0)
Time on NNRTI, median (Q1-Q3); years	6.8 (3.4–9.7)
Time on PI, median (Q1-Q3); years	7.1 (3.9–10.8)
Time on InsTI, median (Q1-Q3); years	3.7 (2.6–4.8)

^a^Only 87 out of 166 were on statins, mainly atorvastatin (*n* = 77)

### HIV Characteristics

A summary of the HIV characteristics of the study participants can be seen in Table 1. Participants had a mean duration of HIV infection of 12.3 years. Median serum levels of CD4 cells were 701 / µL, undetectable viral load was reported by 99% of the persons. Overall, participants had been receiving cART for a median of 9.5 years.

### Cardiovascular risk factors and cardiovascular risk estimation

In our population, hypercholesterolemia and smoking were the most (and equally) prevalent CVRF (36%) followed by hypertension (18%), hypertriglyceridemia (16%), family history of CVD (9%) and type 2 diabetes mellitus (6%). Obesity was present in 6.7% of study participants.

Mean 10-year score in the VASI study was 5.7% using the FHS and 3.3% using the calibration for Spain REGICOR [22]. Cardiovascular risk factors and estimations are summarized in Table 2.

**Table 2:** Cardiovascular risk factors and estimation

CV Risk factors		*N* = 463
BMI, mean ± SD (kg/m^2^)		24.7 ± 3.6
Normal (< 25)		262 (56.6%)
Overweight [25–30)		170 (36.7%)
Obesity (≥ 30)		31 (6.7%)
SBP, mean ± SD (mmHg)		122.9 ± 13.2
DBP, mean ± SD (mmHg)		75.7 ± 8.7
Smoking habit, *N* (%)	Nonsmoker	198 (43.0)
	Active smoker	168 (36.4)
	Ex-smoker	95 (20.6)
Physical activity^a^		79 (26.9)
Family history of CV disease		39 (8.8)
CV disease risk estimation:		
European SCORE equation	Low risk (< 5%)	299 (86.7)
	Intermediate risk (≥ 5–10)	44 (12.8)
	High risk (≥ 10–20%)	2 (0.6)
D:A:D risk equation, *N* (%)	• Low (< 0%)	7 (1.5)
	• Mild (0–4%)	310 (68.0)
	• High (≥ 5%)	139 (30.5)
REGICOR^b^ (%), mean ± SD		3.3 ± 2.2
FRAMINGHAM (%), mean ± SD		5.7 ± 5.4

^a^Physical activity: moderate exercise 30 min/day for 5 days/week or intense exercise 15 min/day for 5 days/week

^b^REGICOR risk classification: High (≥ 10%), intermediate (≥ 5%—< 10%), Low (< 5%)

### Subclinical atherosclerosis: distribution and extension

The primary outcome was to describe the proportion of persons with subclinical atherosclerosis (carotid and iliofemoral territories). Subclinical atherosclerosis was found in 197 participants (42.5%; CI 95% [38.0–47.2]). Prevalence tended to be numerically higher in women (50.7%) than in men (41%) but the difference did not achieve statistical significance (*p* = 0.126). The disease was found more frequently in the femoral arteries (37.8%) than in the carotid vascular bed (18.6%).

**Fig. 1:**
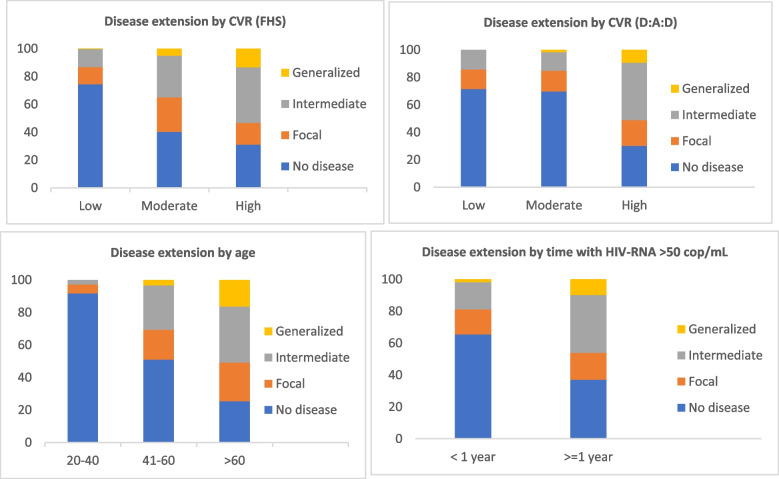
Subclinical atherosclerosis extension by CVR estimation, Age and Cumulative time with HIV-1RNA > 50 cop/mL

The extension of the disease was classified as the number of vascular sites affected (0 to 4). We classified participants as having no disease (no vascular sites affected), focal disease (1 vascular site affected), intermediate disease (2 or 3 sites affected) and generalized disease (4 sites affected). Among participants, focal disease was found in 16%, intermediate disease in 22.5% and generalized disease in 4.1%. We did not find different patterns of disease extension between men and women. Disease extension positively correlated with older age, higher CVR estimation and longer time with detectable viral load (Fig. 1).

### Factors associated with the presence of subclinical atherosclerosis

The association of several HIV and non-HIV related factors with the presence of subclinical atherosclerosis was explored with a bivariate approach. The full results of this analysis can be seen in Table 3. Key non-HIV related factors associated with the presence of subclinical atherosclerosis included older age (OR 1.13, 95%CI 1.10 – 1.15), hypertension (OR 3.52, 95% CI 2.12 – 5.82), smoking habit (OR 4.79, 95% CI 3.17 – 7.26) and diabetes (OR 3.44, 95% CI 1.48 – 8.04). Among the HIV-factors analyzed, those found to be associated with the presence of atherosclerotic plaques included months from HIV diagnosis (OR 1.008, 95% CI 1.006 – 1.010), CDC stage 3 (OR 2.24, 95% CI 1.45 – 3.44), time with HIV-1 RNA > 50 cop/mL (OR 3.24, 95% CI 2.12 – 4.94), VACS score (OR 1.07, 95% CI 1.06 – 1.09), time on antiretroviral therapy (OR 1.15, 95% CI 1.09 – 1.20) and having received protease inhibitors (OR 2.45, 95% CI 1.67 – 3.60). On the contrary, nadir CD4 and current CD4/CD8 ratio were inversely associated (see Table 3).

**Table 3:** Association of HIV and non-HIV-Factors with presence of atherosclerotic plaques (Univariate analysis)

*HIV Factors*	**OR**	**CI 95%**		***p*-value**
Time to diagnosis (months)	1.008	1.006	1.010	0.000
CD4 nadir (cells/µL),	0.998	0.997	0.999	0.000
Current CD4/CD8,	0.630	0.417	0.950	0.027
Cumulative time with HIV-1 RNA > 50 cop/mL (≥ 1 year vs. < 1 year)	3.238	2.124	4.938	0.000
CDC status C (yes vs. no)	2.238	1.454	3.443	0.000
VACS Index	1.074	1.055	1.094	0.000
Time receiving ARV therapy (years)	1.145	1.090	1.203	0.000
History of use of NRTIs therapy (yes vs. no)	0.453	0.146	1.405	0.170
History of use of NNRTIs therapy (yes vs. no)	0.868	0.600	1.255	0.451
History of use of PIs therapy (yes vs. no)	2.452	1.673	3.595	0.000
History of use of InsTI therapy (yes vs. no)	0.773	0.529	1.131	0.185
***Non-HIV factors***	**OR**	**CI 95%**		***p*-value**
Age (years)	1.125	1.097	1.154	0.000
Sex (Male vs. Female)	1.477	0.895	2.439	0.127
Race (Caucasian vs. Other)	0.347	0.182	0.663	0.001
BMI (kg/m^2^)	0.974	0.924	1.026	0.321
SBP (mmHg)	1.025	1.010	1.040	0.001
DBP (mmHg)	1.052	1.029	1.076	0.000
High blood pressure	3.516	2.123	5.821	0.000
Smoking habit (Never vs. Current/former)	4.793	3.165	7.257	0.000
Physical activity	0.416	0.236	0.734	0.002
Total cholesterol (mmol/L)	0.995	0.990	1.000	0.038
HDL (mmol/L)	0.989	0.976	1.003	0.116
LDL (mmol/L)	0.995	0.989	1.001	0.125
Diabetes	3.442	1.475	8.037	0.004
CV risk score (≥ 5% vs. < 5%)	4.257	2.038	8.892	0.000
D:A:D risk equation(≥ 5% vs. < 5%)	5.317	3.444	8.207	0.000
hsCPR (mg/L)	0.949	0.606	1.486	0.820
IL-6 (pg/mL)	0.959	0.807	1.140	0.636
Dimer-D (ng/L)	1.001	1.000	1.001	0.255
CD14 (pg/mL)	1.000	1.000	1.000	0.836
CD163 (pg/mL)	1.000	1.000	1.000	0.639

A multivariate analysis was performed to describe what HIV-related and non-HIV related factors might be associated with the presence of atherosclerotic plaques. The only two explanatory factors that were statistically associated with the presence of atherosclerotic plaques were smoking (OR 5.47, 95% CI 3.36 – 8.90) and age (OR 1.13, 95%CI 1.10 – 1.19).

### Biomarkers sub-study

Among all the study participants, 127 consented to participate in the biomarker sub-analysis. Analyzed biomarkers included serum levels of hsCRP, IL-6, D-Dimer and soluble CD14 and CD163. None of them were associated with the presence of atherosclerotic plaques (Table 3).

### Characteristics of the atherosclerotic plaques

**Fig. 2:**
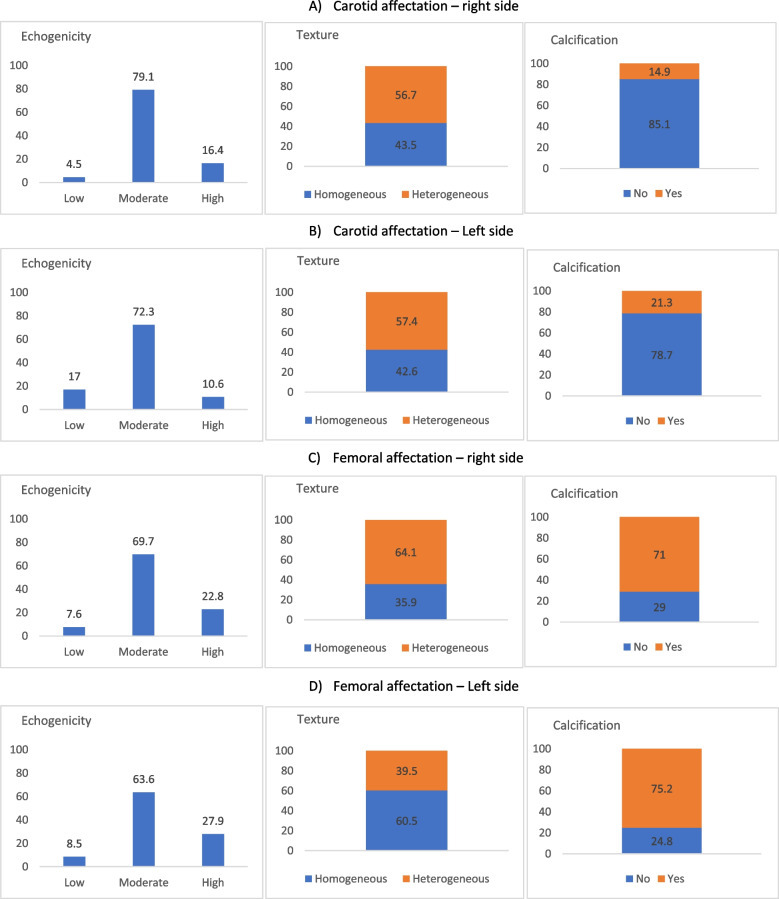
Characteristics of the atherosclerotic plaques

Atherosclerotic plaques were characterized depending on their echogenicity, texture and calcification in both carotid and femoral territories (Fig. 2). Moderate echogenicity was predominant for femoral and carotid territories in both left and right sides. The percentage of patients with carotid plaque homogeneous texture and heterogeneous texture was very similar, in both, left and right side. The percentage of plaque calcification at carotid territory was very low (right side: 14.9% and left side: 21.3%), however, femoral plaques presented a higher percentage of calcification (right side:71.0% and left side: 75.2%).

Intima media thickness mean value was 0.66 ± 0.17 mm.

## Discussion

Several studies have described a very high prevalence of subclinical atherosclerosis among otherwise healthy population [18, 19]. Given the fact that HIV infection is considered a major risk factor for developing cardiovascular disease [23] we initially hypothesized that the prevalence of subclinical atherosclerosis in the HIV population might be even higher. However, the data from our cohort suggest that PLWH has a similar prevalence of subclinical atherosclerosis than does the non-HIV population. An explicit limitation of our study is that we do not have a non-HIV control group to compare. Therefore, any comparison we can make with the general population is approximate. However, the absolute values are not that different from those observed in the general population. So, to conclude that PLWH have a higher number of atherosclerotic plaques, it would be necessary to conduct a study with an appropriate control group.

The impact of cART on the development of CVD in PLWH is a matter of debate. There is reliable evidence regarding the influence of abacavir, mainly when used recently, but the data on the association with protease inhibitors (PI) is controversial. It is difficult to determine the specific role of individual drugs since they are always used in combination. Our study found an association between the use of PI and the presence of plaques but does not have information about the use of specific drugs, which is a limitation of the study. To obtain a definitive analysis of this association, a much larger sample size would be needed, which is not possible with the resources we have available.

Since 1998 when the initial reports of severe premature atherosclerosis in HIV subjects started to appear [24], many studies had concluded that subclinical atherosclerosis is more frequent in PLWH in comparison with non-HIV persons. Many of these studies, however, did only measure the presence of atherosclerotic plaques in a single vascular bed (i.e., the carotid artery) [25–29]. Today, it is well known that this silent disease is not a focal one and that it is more frequently detected in the ileo-femoral territories [18, 19]. In our opinion, prevalence studies should include these vascular beds among others because, the aforementioned conclusion, might be biased by the fact that they are only considering part of the problem.

Studies in the HIV population that have taken into consideration the ileo-femoral territory are scarce. Fernández Soto J et al. did measure the subclinical atherosclerosis in 183 PLWH finding a prevalence higher than ours (62.9% versus 42.5%). Differently from many other studies in HIV and non-HIV populations (including ours), they found a much higher prevalence in the carotid vascular bed (52%) than in the femoral one (36%). Many factors might account for the differences among our two studies. Differently from our cohort, they only recruited persons with a history of HIV-infection of 10 years of duration or longer. The prevalence of some major CVRF in their cohort is much higher (e.g. active smokers 86% vs. 36% or hypertension 36% vs. 18%). Finally, the smaller number of persons analyzed (183 versus 463) might have led to a less accurate point estimation of the prevalence and the 95% confidence intervals for their estimations are not reported in their article [30].

Independently, Protogerou AD et al., evaluated the additive value of femoral ultrasound in a diverse cohort of persons that included 133 PLWH. Unfortunately, in this research paper, the prevalence of subclinical atherosclerosis is not reported separately for PLWH but in a pool of subjects including persons with rheumatoid arthritis and type 2 diabetes mellitus as well [31].

It is commonly accepted that cardiovascular disease is more prevalent in PLWH than in the general population of the same age [10, 11]. Our data do not suggest that a higher prevalence of subclinical atherosclerosis is the reason behind this fact. If atherosclerotic plaques in HIV persons could be more vulnerable and prone to rupture is unknown but could be a feasible explanation that deserves further research. There is a vast body of literature showing a higher prevalence of non-calcified plaques among PLWH when compared with uninfected persons.

In the multicenter AIDS cohort study (MACS), Post WS et al. described a prevalence of coronary atherosclerosis of 77.4% in a group of 618 men living with HIV versus 74.4% in a group of 383 seronegative controls who underwent contrast enhanced cardiac CT. The prevalence of non-calcified plaques was 63.3% in the HIV group versus 53.1% in the uninfected control group. The higher prevalence of non-calcified plaques among the HIV group remained significant after adjusting for age, race, center, cohort and CAD risk factors [32].

In a meta-analysis of observational studies including 6,699 PLWH and 4,168 controls C. Soares et al. described a similar combined prevalence of CAC and coronary plaque in HIV-positive versus HIV-negative participants. Again, a higher pooled prevalence of non-calcified plaque was found in the HIV-positive participants [49% (95% CI, 47%– 52%)] versus the HIV-negative controls [20% (95% CI, 17%—23%)] [33]. Within our participants, we were able to find signs of calcification in most of the plaques in the ileo-femoral vascular bed. However, calcification was much less common in the carotid vascular bed.

Finally, in a substantial proportion of a group of apparently healthy non-HIV subjects, it has been described short-term progression (3 years) of subclinical atherosclerosis (either new onset or baseline disease progression) [34]. In PLWH traditional cardiovascular risk estimations do not predict accurately disease progression [35]. Further studies with a comparative and longitudinal approach will be needed to understand whether atherosclerosis do progress at a similar speed in PLWH. Unfortunately, due to the cross-sectional nature of our investigation, we have not been able to answer this important question.

In conclusion, the proportion of patients with atheromatous plaques detected by vascular ultrasound in our group of PLWH is substantial, but not very different as described in the general population in other studies. Vascular ultrasound constitutes an easy to apply and non-invasive technique that can help to better refine the risk of CVD in PLWH.

In addition to those commented above, our research has some additional limitations. Firstly, as a single center study, the external validity of our conclusions is compromised. Study sample has not been selected randomly so selection biases cannot be disregarded. Finally, our study has the limitations inherent to cross-sectional designs although this kind of design is widely accepted in prevalence studies.

## Data Availability

The datasets used and/or analyzed during the current study are available from the corresponding author on reasonable request.

## Abbreviations

ARV: Antiretrovirals
BMI: Body Mass Index
CAD: Coronary Artery Disease
cART: Combined Antiretroviral Treatment
CDC: Centers for Disease Control and Prevention
CI: Confidence Interval
CVD: Cardiovascular Disease
CVRF: Cardiovascular Risk Factors
DBP: Diastolic Blood Pressure
HIV: Human Immunodeficiency Virus
hsCRP: Highly sensitive C Reactive Protein
IMT: Intima Media Thickness
InsTI: Integrase strand Transfer Inhibitor
MACS: Multicenter AIDS Cohort Study
NNRTI: Non-nucleoside analogue Reverse Transcriptase Inhibitor
NRTI: Nucleoside analogue Reverse Transcriptase Inhibitor
OR: Odds Ratio
PI: Protease Inhibitor
PLWH: People Living With HIV
SBP: Systolic Blood Pressure

## References

[1] Palella FJ Jr, Delaney KM, Moorman AC et al. Declining morbidity and mortality among patients with advanced human immunodeficiency virus infection. HIV Outpatient Study Investigators. N Engl J Med. 1998;338(13):853–60. 10.1056/NEJM199803263381301. 10.1056/NEJM199803263381301 9516219

[2] Samji H, Cescon A, Hogg RS, et al. Closing the gap: increases in life expectancy among treated HIV-positive individuals in the United States and Canada. PLoS One. 2013;8(12):e81355. Available at: http://www.ncbi.nlm.nih.gov/pubmed/24367482. 10.1371/journal.pone.0081355 PMC3867319 24367482

[3] Autenrieth CS, Beck EJ, Stelzle D, Mallouris C, Mahy M, Ghys P. Global and regional trends of people living with HIV aged 50 and over: Estimates and projections for 2000-2020. PLoS One. 2018;13(11):e0207005. 10.1371/journal.pone.0207005. 10.1371/journal.pone.0207005 PMC6264840 30496302

[4] Wandeler G, Johnson LF, Egger M. Trends in life expectancy of HIV-positive adults on antiretroviral therapy across the globe: comparisons with general population. Curr Opin HIV AIDS. 2016;11:492–500. 27254748 10.1097/COH.0000000000000298 PMC5055447

[5] Vance DE, Mugavero M, Willig J, Raper JL, Saag MS.. Aging With HIV: A Cross-Sectional Study of Comorbidity Prevalence and Clinical Characteristics Across Decades of Life. J Assoc Nurses AIDS Care. 2011;22(1):17–25. 10.1016/j.jana.2010.04.002 20471864

[6] Guaraldi G, Orlando G, Zona S, et al. Premature age-related comorbidities among HIV-infected persons compared with the general population. Clin Infect Dis. 2011;53:1120–6. 10.1093/cid/cir627. 21998278 10.1093/cid/cir627

[7] Ekrikpo UE, Kengne AP, Bello AK, et al. Chronic kidney disease in the global adult HIV-infected population: A systematic review and meta-analysis. PLoS One. 2018;13(4):e0195443. 10.1371/journal.pone.0195443. 29659605 10.1371/journal.pone.0195443 PMC5901989

[8] Nanditha NGA, Paiero A, Tafessu HM, et al. Excess burden of age-associated comorbidities among people living with HIV in British Columbia, Canada: a population-based cohort study. BMJ Open. 2021;11(1):e041734. 10.1136/bmjopen-2020-041734. 33419911 10.1136/bmjopen-2020-041734 PMC7799128

[9] Winston A, Spudich S. Cognitive disorders in people living with HIV. Lancet HIV. 2020;7(7):e504–13. 10.1016/S2352-3018(20)30107-7. PMID: 32621876. 32621876 10.1016/S2352-3018(20)30107-7

[10] Alonso A, Barnes AE, Guest JL, et al. HIV infection and incidence of cardiovascular diseases: an analysis of a large healthcare database. J Am Heart Assoc. 2019;8(14):e012241. 10.1161/JAHA.119.012241. 31266386 10.1161/JAHA.119.012241 PMC6662120

[11] Islam FM, Wu J, Jannson J, Wilson DP. Relative risk of cardiovascular disease among people living with HIV: a systematic review and meta-analysis. HIV Med. 2012;13:453–68. 22413967 10.1111/j.1468-1293.2012.00996.x

[12] Triant VA, Lee H, Hadigan C, Grinspoon K. Increased acute myocardial infarction rates and cardiovascular risk factors among patients with human immunodeficiency virus disease. J Clin Endocrinol Metab. 2007;92:2506–12. 17456578 10.1210/jc.2006-2190 PMC2763385

[13] Islam FM, Wu J, Jannson J, Wilson DP. Relative risk of cardiovascular disease among people living with HIV: a systematic review and meta-analysis. HIV Med. 2012;13:453–68. 22413967 10.1111/j.1468-1293.2012.00996.x

[14] Fedele F, Bruno N, Mancone M. Cardiovascular risk factors and HIV disease. AIDS Rev. 2011;13:119–29. 21587343

[15] Duprez DA, Neuhaus J, Kuller LH, Tracy R, Belloso W, et al. Inflammation, coagulation and cardiovascular disease in HIV-infected individuals. PLoS One. 2012;7(9): e44454. 10.1371/journal.pone.0044454. 22970224 10.1371/journal.pone.0044454 PMC3438173

[16] Brenchley JM, Price DA, Schacker TW, et al. Microbial translocation is a cause of systemic immune activation in chronic HIV infection. Nat Med. 2006;12:1365–71. 17115046 10.1038/nm1511

[17] Crowe SM, Westhorpe CL, Mukhamedova N, et al. The macrophage: the intersection between HIV infection and atherosclerosis. J Leukoc Biol. 2010;87(4):589–98. 10.1189/jlb.0809580. 19952353 10.1189/jlb.0809580 PMC3085483

[18] Fernández-Friera L, Peñalvo JL, Fernández-Ortiz A, et al. Prevalence, vascular distribution, and multiterritorial extent of subclinical atherosclerosis in a middle-aged cohort: The PESA (Progression of Early Subclinical Atherosclerosis) Study. Circulation. 2015;131(24):2104–13. 10.1161/CIRCULATIONAHA.114.014310. Epub 2015 Apr 16 PMID: 25882487. 25882487 10.1161/CIRCULATIONAHA.114.014310

[19] Laclaustra M, Casasnovas JA, Fernández-Ortiz A, et al. Femoral and carotid subclinical atherosclerosis association with risk factors and coronary calcium: the AWHS study. J Am Coll Cardiol. 2016;67(11):1263–74. 10.1016/j.jacc.2015.12.056. 26988945 10.1016/j.jacc.2015.12.056

[20] ESC/EAS guidelines for the management of dyslipidaemias. lipid modification to reduce cardiovascular risk. Eur Heart J. 2020;41:111–88. 31504418 10.1093/eurheartj/ehz455

[21] Tate JP, Justice AC, Hughes MD, et al. An internationally generalizable risk index for mortality after one year of antiretroviral therapy. AIDS. 2013;27:563–72. 23095314 10.1097/QAD.0b013e32835b8c7f PMC4283204

[22] Ramos R, Solanas P, Cordón F, et al. Comparison of population coronary heart disease risk estimated by the Framingham original and REGICOR calibrated functions. Med Clin (Barc). 2003;121(14):521–6 article in Spanish. 14599406 10.1016/S0025-7753(03)74007-X

[23] Hsue PY, Waters DD. Time to recognize HIV infection as a major cardiovascular risk factor. Circulation. 2018;138(11):1113–5. 10.1161/CIRCULATIONAHA.118.036211. 30354392 10.1161/CIRCULATIONAHA.118.036211 PMC8063774

[24] Henry K, Melroe H, Huebsch J, Hermundson J, Levine C, Swensen L, Daley J. Severe premature coronary artery disease with protease inhibitors. Lancet. 1998 May 2;351(9112):1328. 10.1016/S0140-6736(05)79053-X. 10.1016/S0140-6736(05)79053-X 9643798

[25] Saumoy M, Di Yacovo S, Pérez S, et al. Carotid atherosclerosis in virologically suppressed HIV patients: comparison with a healthy sample and prediction by cardiovascular risk equations. HIV Med. 2021;22(7):581–91. 10.1111/hiv.13093. 33817938 10.1111/hiv.13093

[26] McLaughlin MM, Ma Y, Scherzer R, et al. Association of viral persistence and atherosclerosis in adults with treated HIV infection. JAMA Netw Open. 2020;3(10):e2018099. 10.1001/jamanetworkopen.2020.18099. 33119103 10.1001/jamanetworkopen.2020.18099 PMC7596582

[27] Calza L, Borderi M, Colangeli V, et al. No progression of subclinical atherosclerosis in HIV-infected patients starting an initial regimen including tenofovir alafenamide/emtricitabine plus raltegravir, dolutegravir or elvitegravir/cobicistat during a two-year follow-up. Infect Dis (Lond). 2020;52(4):249–56. 10.1080/23744235.2019.1707279. 10.1080/23744235.2019.1707279 31876437

[28] Hanna DB, Guo M, Bůžková P, et al. HIV infection and carotid artery intima-media thickness: pooled analyses across 5 cohorts of the NHLBI HIV-CVD Collaborative. Clin Infect Dis. 2016;63(2):249–56. 10.1093/cid/ciw261. 27118787 10.1093/cid/ciw261 PMC4928384

[29] Hulten E, Mitchell J, Scally J, Gibbs B, Villines TC. HIV positivity, protease inhibitor exposure and subclinical atherosclerosis: a systematic review and meta-analysis of observational studies. Heart. 2009;95(22):1826–35. 10.1136/hrt.2009.177774. 19632982 10.1136/hrt.2009.177774

[30] Fernández Soto J, Romero-Jiménez MJ, Alarcón García JC, Bonet Estruch E, Sánchez Ramos JL, Castaño López MÁ. Predictors of subclinical atherosclerosis in HIV. BMC Infect Dis. 2023;23(1):17. 10.1186/s12879-022-07976-1. 36627565 10.1186/s12879-022-07976-1 PMC9832619

[31] Protogerou AD, Fransen J, Zampeli E, Argyris AA, Aissopou E, Arida A, Konstantonis GD, Tentolouris N, Makrilakis K, Psichogiou M, Daikos G, Kitas GD, Sfikakis PP. The additive value of femoral ultrasound for subclinical atherosclerosis assessment in a single center cohort of 962 adults, including high risk patients with rheumatoid arthritis, human immunodeficiency virus infection and type 2 diabetes mellitus. PLoS One. 2015;10(7):e0132307. 10.1371/journal.pone.0132307. 10.1371/journal.pone.0132307 PMC4521696 26230728

[32] Post WS, Budoff M, Kingsley L, Palella FJ Jr, Witt MD, Li X, George RT, Brown TT, Jacobson LP. Associations between HIV infection and subclinical coronary atherosclerosis. Ann Intern Med. 2014;160(7):458–67. 10.7326/M13-1754. 24687069 10.7326/M13-1754 PMC4143766

[33] Soares C, Samara A, Yuyun MF, Echouffo-Tcheugui JB, Masri A, Samara A, Morrison AR, Lin N, Wu WC, Erqou S. Coronary artery calcification and plaque characteristics in people living with hiv: a systematic review and meta-analysis. J Am Heart Assoc. 2021;10(19): e019291. 10.1161/JAHA.120.019291. 34585590 10.1161/JAHA.120.019291 PMC8649136

[34] Lopez-Melgar B, Fernández-Friera L, Oliva B, et al. Short-term progression of multiterritorial subclinical atherosclerosis. J Am Coll Cardiol. 2020;75(14):1617–27. 32273027 10.1016/j.jacc.2020.02.026

[35] Shaikh K, Bhondoekhan F, Haberlen S, Nakanishi R, Roy SK, Alla VM, Brown TT, Lee J, Osawa K, Almeida S, Rahmani S, Nezarat N, Sheidaee N, Kim M, Jayawardena E, Kim N, Hathiramani N, Palella FJ, Witt M, Ahmad K, Kingsley L, Post WS, Budoff MJ. Coronary artery plaque progression and cardiovascular risk scores in men with and without HIV-infection. AIDS. 2022;36(2):215–24. 10.1097/QAD.0000000000003093. 10.1097/QAD.0000000000003093 PMC8702479 34608042

